# Fabrication of a Novel Conductometric Biosensor for Detecting *Mycobacterium avium* subsp. *paratuberculosis* Antibodies

**DOI:** 10.3390/s8096015

**Published:** 2008-09-26

**Authors:** Chika Okafor, Daniel Grooms, Evangelyn Alocilja, Steven Bolin

**Affiliations:** 1 Large Animal Clinical Sciences, Michigan State University, East Lansing, MI, U.S.A., 48824; E-Mail: groomsd@cvm.msu.edu; 2 Biosystems and Agricultural Engineering, Michigan State University, East Lansing, MI, U.S.A., 48824; E-Mail: alocilja@msu.edu; 3 Pathobiology and Diagnostic Investigation, Michigan State University, East Lansing, MI, U.S.A., 48824; E-Mail: bolins@dcpah.msu.edu

**Keywords:** Conductometric, biosensor, immunoassay, electrochemical immunosensor, polyaniline, paratuberculosis, Johne's disease, on-site diagnosis

## Abstract

Johne's disease (JD) is one of the most costly bacterial diseases in cattle. In the U.S., economic losses from the disease have been estimated to exceed $1,500,000,000 per year, mainly from the effects of reduced milk production. Current diagnostic tests for JD are laboratory based and many of those tests require specialized equipment and training. Development of rapid and inexpensive diagnostic assays, which are adapted for point-of-care applications, would aid in the control of JD. In this study, a polyaniline (Pani)-based conductometric biosensor, in an immunomigration format, was fabricated for the detection of serum antibody (IgG) against the causal organism of JD, *Mycobacterium avium* subsp. *paratuberculosis* (MAP). Immobilized *Mycobacterium avium* purified proteins in the capture membrane were used to detect MAP IgG, previously bound with Pani/anti-bovine IgG* conjugate in the conjugate membrane. After detection, the Pani in the sandwiched captured complex bridges an electrical circuit between the silver electrodes, flanking the capture membrane. The electrical conductance, caused by Pani, was measured as drop in electrical resistance. Testing of the biosensor with known JD positive and negative serum samples demonstrated a significant difference in the mean resistance observed between the groups. This proof-of-concept study demonstrated that a conductometric biosensor could detect MAP IgG in 2 minutes. The biosensor's speed of detection and the equipment involved would, among other things, support its application towards the various point-of-care opportunities aimed at JD management and control.

## 1. Introduction

Johne's disease (JD) is a chronic gastrointestinal disease of ruminants caused by *Mycobacterium avium* subsp. *paratuberculosis* (MAP). JD causes significant economic losses in the cattle industry. In the U.S., economic losses from the disease have been estimated to exceed $1,500,000,000 per year [1], mainly from the effects of reduced milk production [2]. Additional sources of losses are unrealized income related to premature culling of cattle, reduced meat quantity at slaughter and animal death. Although there is evidence that MAP may be associated with Crohn's disease in humans, MAP is not currently recognized as a zoonotic pathogen [3]. Economic losses from JD and the concern that MAP may be a zoonotic pathogen have increased the urgency to control the spread of MAP in domestic animals.

Effective control of JD has been challenging. Limitations in currently available diagnostic tests contribute to this challenge. Diagnosis of JD is aimed at detecting MAP or its DNA in feces, tissues, and occasionally milk; or by detecting an immune response against MAP. Currently, bacterial culture is most commonly used in MAP detection [4]. Other commonly used methods to detect MAP or to detect infection with MAP include polymerase chain reaction (PCR) for detection of MAP DNA [5] and enzyme-linked immunosorbent assay (ELISA) for detection of antibody against MAP (IgG) [6]. However, bacterial culture is expensive and requires 7-12 weeks for completion [7, 4]; PCR and ELISA require specialized equipment and training. These currently used diagnostic tests may not be easily adapted for on-site diagnosis and are not readily accessible to some developing countries. The development of new JD diagnostic assays, which are adaptable to the field and are potentially useful in point-of-care applications, would be beneficial in furthering JD control efforts.

Biosensors are among the new growing pathogen detection or disease diagnostic assays. A biosensor is an analytical device that contains a transducer, integrated with or placed close to a biological sensing element (BSE) (i.e. antibody) such that a specific biological recognition (i.e. antigen-antibody binding) reaction produces a measurable signal change in a physicochemical detector component [8]. A biosensor can be classified based on either the BSE or the transducer components and sometimes a combination of both. Examples of classification based on BSE include antibody-based, DNA-based, enzyme-based, and antigen-based biosensors. Examples of classification based on transducer include resonant, optical, thermal, ion-sensitive field effect transistors (ISFETs), and electrochemical biosensors. Electrochemical biosensors are further classified as amperometric, potentiometric, and conductometric biosensors.

A conductometric biosensor measures electrical conductance/resistance as its signal change. There has been a considerable interest in using conductive polymers (polyaniline, polypyrrole, polyacetylene, and polythiophene) in the development of conductometric biosensors [9, 10]. Conductive polymers are transducers in conductometric biosensors. Polyaniline (Pani) has been among the most extensively used conductive polymers, due to its strong bio-molecular interactions [11], excellent environmental stability, and good conductivity [12]. In a conductometric biosensor, Pani is placed close to or integrated with the biological element (i.e. antibody) such that Pani relays any antigen-antibody binding as a measured electrical quantity. With increased necessity for rapid detection assays in recent times, conductometric biosensors have been applied in various biological and biomedical sciences. The applications include determination of glucose and urea in blood [13], heavy metal ions and pesticides in water [14], and detection of *E. coli* O157:H7 [15], *Bacillus cereus* [16], and Bovine viral diarrhea virus [17]. However, this relatively new assay has not been applied towards JD diagnosis. The development of a biosensor as a rapid, inexpensive, miniaturized, and field-based JD diagnostic assay would support more frequent and widespread testing of animals.

The objective of this study was to fabricate and test a conductometric biosensor for detecting IgG in sera from cattle that reacts with MAP. The basic architecture of the biosensor is based on previous publications [15-17], however the detection principle is a unique variation. Unlike the previous biosensors, which were fabricated to detect bacterial or viral organisms [15-17], the biosensor in this study was designed to detect antibodies to a bacterium. Optimization of the fabricated biosensor for JD diagnosis would support various point-of-care applications and frequent testing of animals especially at the point-of-sale, thus guiding the making of management decisions that would improve JD control.

## 2. Results

### 2.1. Preliminary testing of biosensor with purified serum

**Figure 1.**
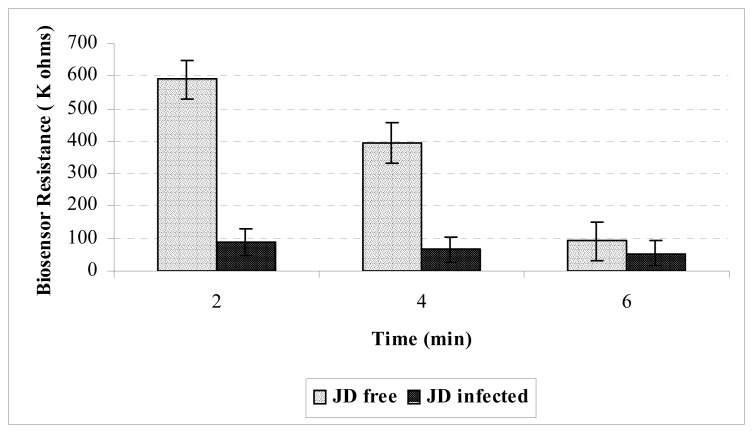
Conductometric biosensor response on purified IgG from serum of JD-infected and JD-free cattle (eight replications per experimental set).

A preliminary testing result of a JD-infected animal against a JD-free animal, with ELISA OD values of 3.270 and -0.048 respectively, is shown in Figure 1. For each time interval, significant lower resistant values were recorded for the JD-infected animal as compared to the JD-free animal.

### 2.2. Testing of biosensor with unpurified serum

Results for each sample tested in the biosensor at different time points are shown in Table 1. The conductometric biosensor evaluation of the samples showed that at each 2-minute interval, the JD positive samples had numerically lower resistance values than the JD negative samples.

**Figure 2.**
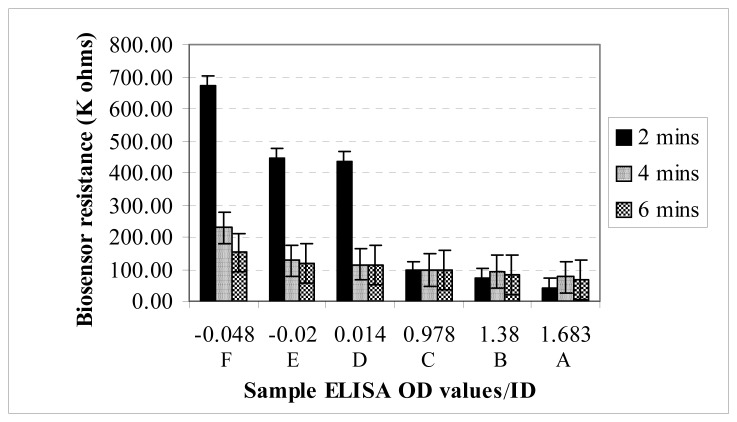
Influence of biosensor reading time and ELISA OD on the biosensor resistance; ELISA OD < 1 = JD negative, >1 = JD positive.

A 2-way ANOVA showed that the difference in mean resistance values of the samples was significantly affected by the ELISA OD values, the reading times, as well as their interactions (P <0.05) (Figure 2). Hence, the effect of ELISA OD values on the biosensor resistance depended on what time the reading was taken.

The Holm-Sidak test showed that at 2 minutes, the mean resistance value of each of the JD positive samples (A, B, C) was significantly different (P <0.05) from the mean resistance of each of the JD negative samples (D, E, F). At 4 minutes, the difference in mean resistance was statistically significant only between sample A and sample F. And at 6 minutes, the difference in mean resistance between the JD positive samples and the JD negative samples was not statistically significant. The intra-assay coefficient of variation of the biosensor at 2 minutes was 14.48%.

## 3. Discussion

In this study, a conductometric biosensor was successfully designed and fabricated for detecting MAP IgG in serum, containing both MAP and non-MAP IgG. At the conjugate membrane, both JD negative and JD positive samples potentially formed Pani-AB/IgG*-IgG complex, but the difference in resistance values showed that among JD positive samples (ELISA OD >1), more Pani-AB/IgG*-IgG complexes were bound at the capture membrane. The immobilized complexes could be attributed to the ability of the MAPPD (MAP antigen) on the capture membrane to bind with MAP IgG in the positive samples while IgG in JD negative samples continued unbound to the absorption membrane. Due to its conductive property, the polyaniline in the Pani-AB/IgG*-IgG complex caused a lower electrical resistance in JD positive samples. Generally, the biosensor electrical resistance decreased as the ELISA OD increased from JD negative to JD positive samples. Although not specifically addressed in this study, the relationship between the ELISA OD (relative concentration of MAP antibodies) and the biosensor values (Figure 2) suggest that the biosensor results could be used to quantify ELISA OD.

The difference in biosensor values was statistically significant at 2, but not at 4 and 6 minutes. It is not clear why the resistance in negative samples dropped after 2 minutes. One possibility is the absorption membrane's inability to completely pull Pani-AB/IgG*-IgG complex from the capture membrane. In this study, an approximate time of 2 minutes elapsed before the serum (0.1ml) was pulled to the absorption membrane by capillary action. However, as time passed by, the capture channel was progressively clogged with the subsequent flow of Pani-AB/IgG*-IgG complex. At this time, it appeared that the absorption membrane was not able to pull completely the complexes from the capture membrane. The clogged Pani-AB/IgG*-IgG complex could have been responsible for the lowered resistance values obtained after 2 minutes. This could explain why there was no significant difference in mean resistance values of the samples at 4 and 6 minutes.

Another issue was that the conductometric biosensor had high variance within each sample. The biosensor's intra-assay coefficient of variation (%CV) at 2 minutes was 14.48%. A reasonable target for %CV in routine diagnostic testing is 10-15% but a value of 10% or less is considered satisfactory [18]. The free-hand application of silver electrodes on the capture membrane introduced variability in the width of the capture channels from one biosensor strip to the other. Among JD positive samples, the ease at which Pani forms an electrical bridge on the capture membrane would depend on the width of the channel; narrower channels would produce stronger electrical conductance than wider channels. The non-uniform channels could also introduce variability in fluid flow. Such variability in the biosensor's channels while testing the same sample, could affect the result obtained within each sample and may be responsible for the high variance within tests in the biosensor. A possible way to limit this variability is to have a uniform screen-printing of silver electrodes on the capture membrane. Other potential sources of variability include choice of MAP antigen, MAPPD concentration, AB/IgG* concentration, and polyaniline concentration. Future optimization research would address these variability sources.

The long term applications of the conductometric biosensors include miniaturization into disposable test kits for JD diagnosis; a prerequisite test for animals at point-of-sale; the adaptation of the assay for multiplex testing of pathogens like BVDV, Bovine leukosis virus, *E. coli*, etc; and automation for in­line testing of milk samples for JD in dairy farms. The assay could be developed into an equivalent of ELISA 96 well plates, such that larger sample numbers could be analyzed in minutes. The attributes of the conductometric biosensor support its applications to numerous diseases of veterinary and public health concern, especially the emerging diseases, and would improve food animal defense.

## 4. Materials and Methods

The biosensor used in this study comprises an immunosensing component and a signal detector system. The immunosensing component of the biosensor consists of four individual membranes: sample application, conjugate, capture, and absorption membranes (Hi-Flow Plus Assembly Kit, Millipore, Bedford, MA, U.S.A.). These membranes were prepared, fabricated, and assembled to form a functional biosensor. The choice of these membranes was based on previous studies [10, 15, 19]. The sample application membrane, made of cellulose, provides a quick flow of the sample with no or minimal interference; the conjugate membrane, made of fiberglass, adsorbs the polyaniline-conjugated antibody and allows easy flow of fluid; the pore size of the capture membrane, made of nitrocellulose, allows the flow of non-target molecules while providing good adsorption properties for the immobilized molecule; and the absorption membrane, a cellulose membrane, absorbs and retains the fluid from the capture membrane. For a functional conductometric biosensor, the capture membrane is printed with silver electrodes, yielding a 1 mm wide capture channel. The electrodes are connected to an etched copper wafer, and finally the wafer is connected to the signal detector system, an ohmmeter. Silver electrodes and the etched copper wafer has been demonstrated to possess good electrical and easy fabrication properties [10, 15, 19].

### 4.1. Capture membrane preparation

The capture membrane was prepared at room temperature under a clean biosafety cabinet unless otherwise stated. The capture membrane was first flushed with distilled water, to remove any debris, and air-dried for 0.5 h. To activate the membrane surface, it was flushed with 10% methanol and air-dried for 0.5 h. To provide a crosslink between the nitrocellulose membrane and the biological receptor molecule, the membrane was washed with 0.5 % glutaraldehyde solution (1.2 mL) and air-dried for 1 h. A total volume of 1.2 mL of 1 mg/mL *Mycobacterium avium* purified protein derivative (MAPPD) (AntelBio, East Lansing, Michigan) was pipetted on the membrane. The membrane was placed in a closed plastic container and incubated (Isotemp incubator, Fisher Scientific) at 35°C for 1 h. MAPPD was used as the antigen because *Mycobacterium avium* is antigenically similar to MAP. Afterwards, the membrane was washed with 1.2 mL of 0.1 M Tris buffer containing 0.1% (v/v) Tween-20, to remove all non-specifically absorbed MAPPD. Finally, the membrane, placed in a closed plastic container, was incubated at 35°C for 0.75 h, air-dried for 0.5 h, and was set to be fabricated.

### 4.2. Polyaniline–Anti bovine IgG conjugation

AquaPass polyaniline (Mitsubishi Rayon Co, Japan) was diluted to 0.001 % with 0.1 M phosphate buffer solution. Purified mouse clone BG-18 monoclonal anti-bovine IgG (Sigma-Aldrich, St Louis, Missouri) was added to the 0.001 % AquaPass polyaniline (Pani) solution to produce a final monoclonal anti-bovine antibody (AB/IgG*) concentration of 0.0115 mg/ml. To form Pani-AB/IgG* conjugate, 4 ml of the AB/IgG* solution was left to conjugate with the Pani in a hybridization oven at 27°C for 1.0 h. Afterwards, 0.5 ml of 0.1M Tris buffer containing 0.1 % casein (pH 9.0), a blocking agent, was added to the Pani-AB/IgG* conjugate solution and left to react in a hybridization oven at 27°C for 0.5 h.

### 4.3. Conjugate membrane immobilization

To immobilize Pani-AB/IgG* conjugate on the conjugate membrane, a conjugate membrane was immersed in the Pani-AB/IgG* conjugate solution until saturated and then air-dried at room temperature under a clean biosafety cabinet for 0.75 h.

### 4.4. Immunosensor fabrication

The capture membrane, besides the prepared portion at the center, has waterproof adhesives at both ends, and provides the backing for attachment of the other immunosensing membranes. These waterproof adhesives were peeled and the other membranes were attached to the waterproof ends during fabrication.

**Figure 3.**
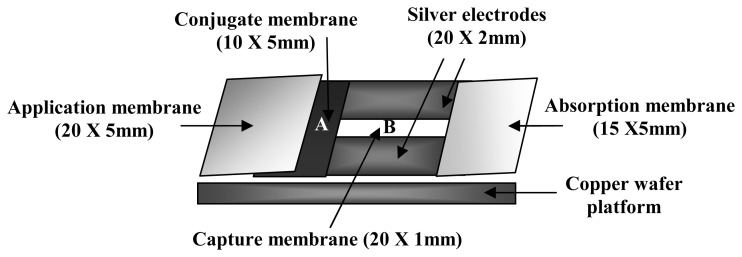
Schematic of the fabricated immunosensor strip **(A)** conjugate membrane containing Pani-AB/IgG **(B)** capture membrane with immobilized MAPPD.

First, the conjugate membrane was attached to one end of the prepared portion of the capture membrane, and then the application membrane was attached overlaying a portion of the conjugate membrane. The absorption membrane was attached on the opposite end of the capture membrane, to complete the immunosensor fabrication. The fabricated immunosensor was cut into 5 mm wide immunosensor strips. With a silver-microtip conductive pen (MG Chemicals, Surrey, B.C., Canada), silver electrodes were hand-printed on both sides of the capture membrane, such that an approximate 1 mm wide capture channel was produced (Figure 3).

### 4.5. Conductometric biosensor assembly

Each silver electrode, flanking the capture membrane, was connected to a copper wafer (Figure 3); connection was hand-printed with a silver-microtip conductive pen. The two ends of the copper wafer were connected to an ohmmeter (Model: 2880A BK Precision multimeter, Worchester, MA, U.S.A.).

### 4.6. Samples

The developed biosensor was tested with both JD positive and JD negative bovine serum samples. The positive samples were collected from clinical JD cows housed at the Michigan State University Veterinary Research Farm, while the negative samples were collected from cows at the Michigan State University Dairy Teaching and Research Center, who had been tested negative for JD a minimum of three times. JD status of the samples was determined by a commercially available MAP ELISA (PARACHEK, Prionics, Schlieren-Zurich, Switzerland), performed at the Diagnostic Center for Population and Animal Health, Michigan State University. The ELISA interpretation was based on the optical density (OD) values, a reflection of the MAP antibody concentration in each sample. ELISA OD values < 1.0 are considered JD negative and > 1.0 are considered JD positive.

### 4.7. Principle of conductometric biosensor detection

**Figure 4.**
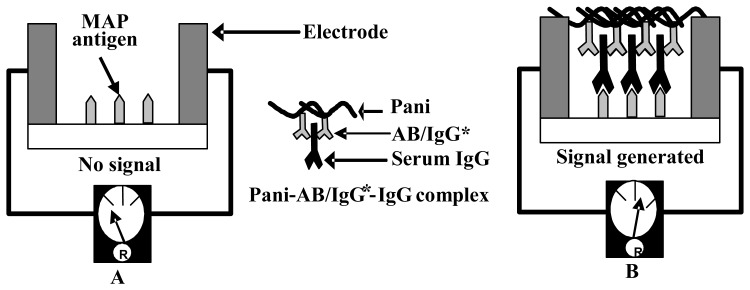
Cross-section of a capture membrane **(A)** before **(B)** after positive JD sample application.

One hundred microliters of sample is applied to the application membrane and is drawn into the entire channel of the immunosensor strip by capillary action. The sample passes the conjugate membrane, where serum IgG, both MAP and non-MAP, are bound to the Pani-AB/IgG* conjugate, forming Pani-AB/IgG*-IgG complex (Figure 4). The complex is drawn into the capture membrane, where immobilized MAPPD captures the MAP specific IgG (JD positive serum) and allow the non-MAP IgG to flow to the absorption membrane. As more and more MAP IgG are captured, the Pani in the Pani-AB/IgG*-IgG complex forms a bridge between the silver electrodes, flanking the capture membrane. Pani causes an electrical conductance through the electrodes; a higher electrical conductance is recorded as a reduced resistance.

### 4.8. Sample testing and signal measurement

Preliminary testing was conducted using purified serum of animals tested to be JD positive and JD negative. The serum was purified with Melon™ gel IgG purification kit (Pierce Biotechnology, Rockford, IL, U.S.A.), according to the manufacturer's specification. The purification step was aimed at removing the non-relevant serum proteins that could compete with IgG during Pani conjugation. Subsequent testing was aimed at verifying the response of the biosensor with unpurified field samples, which meets the objectives of developing a non-laboratory based assay. After each sample application, the resistance value (Kiloohms) was recorded at 2, 4, and 6 minutes. Eight replications were performed for each sample in the preliminary testing while three replications were performed on each sample of the subsequent testing. The mean values of each sample and their standard deviations were calculated.

### 4.9. Statistical analyses

A 2-way ANOVA was used to analyze if the mean resistance values were significantly different among the sample groups, adjusting for the effects of different ELISA OD values and different reading times. Holm-Sidak test was used to isolate which group(s) differed from the others. The statistical analyses were performed with SigmaStat 3.1 software. Intra-assay coefficient of variation of the biosensor was calculated to evaluate the precision of the biosensor assay.

## 5. Conclusions

The fabricated conductometric biosensor developed in this study could differentiate between the relative MAP antibodies concentration of JD positive and negative samples in 2 minutes. The assay was portable for adaptation in on-site diagnosis and could support animal point-of-sale testing, routine herd testing, and clinical animal testing, especially in places with limited access to the currently available JD diagnostic tests. Such test results will provide a basis for timely management decisions that would control the spread of JD among animals. Hence, a conductometric biosensor is a promising diagnostic assay that could improve JD control. Further studies would explore the optimization of the biosensor and testing with larger sample sizes.

## Figures and Tables

**Table 1.** Conductometric biosensor analysis of bovine serum samples at 3 time intervals.

**Sample ID**	**Sample ELISA OD values**	**Conductometric biosensor mean (n=3) resistance** **(Kilo ohms) at 3 time intervals**		

		**2 min** **Mean ± SD**	**4 min** **Mean ± SD**	**6 min** **Mean ± SD**

A	1.683**	43.47 ± 4.76^a^	75.63 ± 32.20^a^	66.63 ± 24.66^a^
B	1.380**	70.33 ± 3.95^a^	93.43 ± 33.50^ab^	81.20 ± 33.98^a^
C	0.978**	95.43 ± 12.58^a^	97.60 ± 30.19^ab^	97.13 ± 24.94^a^
D	0.014*	437.00 ± 33.29^b^	114.73 ± 23.97^ab^	112.83 ± 20.87^a^
E	-0.020*	448.37 ± 99.41^c^	125.83 ± 19.69^ab^	117.73 ± 20.85^a^
F	-0.048*	672.33 ± 101.93^c^	228.53 ± 162.9^b^	152.13 ± 20.33^a^

** Johne's disease (JD) positive,

* JD negative, the negative OD values are from samples that have less background optical density than the negative serum control,

SD = standard deviation Different superscripts a b c within columns indicate significant differences between the mean resistance of the samples (p <0.05).
